# The Medicinal Properties and Use of Iodine

**Published:** 1855-07

**Authors:** F. R. Payne

**Affiliations:** Marshall, Ill.


THE NORTH-WESTERN

### MEDICAL AND SURGICAL JOURNAL.

NEW SERIES.

VOL. IV. JULY, 1855. NO. 7.

### ORIGINAL COMMUNICATIONS.

ARTICLE I.—*The Medicinal properties and use of Iodine.*—Read before the Æsculapian Society, May, 1855, by F. R. Payne, M. D., Marshall, Ill.

We live in an age of improvement, at least one that surpasses all others in activity, enterprise, and innovation. Among these evidences of intelligence and industry, the Profession of Medicine is far from being in the back ground. Within the last few years we have had several valuable accessions to our Science, and we have every reason to believe that the spirit of inquiry which led to these discoveries will not die out when the present generation has passed away. My attention has for many years been directed to the Medicinal properties of Iodine, and the object of this paper will be fully attained should I be successful in eliciting a willingness on the part of my professional brethren (who are more highly favored than myself) to more thoroughly test the curative properties of this valuable remedy. It has been an agent that has long been known to exert a powerful and extensive influence on the animal economy, consequently we cannot at this time refer to all the indications for its use.

Perhaps no country practitioner has used it more extensively than I, and my suggestions are based upon my experience in the use of the remedy. Iodine in small doses is a tonic—it increases the appetite and improves faulty nutrition. It also exerts a powerful influence upon the absorbents and glandular system. Being satisfied that it possessed this power in an eminent degree, I have been induced to test its virtue in the cure of incipient phthisis pulmonalis. Many remedies have been suggested for the cure of this formidable malady, but all in turn have been abandoned and the profession generally induced to believe that no remedy yet discovered could be relied on in the treatment of this disease.—Recently, however, there are found some eminent physicians who firmly believe that phthisis pulmonalis may occasionally, in one stage or another, be cured. If it can be successfully treated in one case, or even a spontaneous cure is effected, have we not reason to hope for a similar result in many cases. Should we be able to discover a remedy that will excite the absorbents to such a degree as to remove tuburculous matter—subdue the diathesis, and by so doing prevent the deposition of other tubercles, then will we have arrived at an important period in the history of medicine,—then will thousands of the most gifted and beautiful of our race be saved from a premature grave.

As evidences of the curability of incipient phthisis pulmonalis, I desire to introduce a brief synopsis of two cases successfully treated with with Iodine.

Mr. L., age 64 years, on the 15th day of November, 1853, labored under the following symptoms: Pain in the breast referred to the apex of left lung, more or less cough, particularly when lying down and in the morning, expectoration of a tough mucus consistence, and but little thrown up, fever with regular exacerbations followed with night sweats, gradual emaciation without sufficient visible cause, appetite capricious,—patient had for some days been troubled with diarrhœa. The digestive organs did not perform their healthy functions. He was very ingenious in presenting arguments to convince me that his lungs were not seriously affected. He was able to be up part of the day, but could not endure much exercise.

The physical signs were such in connection with the rational evidence in the case, as to induce me to believe that it was an unmistakable case of incipient phthisis pulmonalis. At the apex of the left lobe of the lungs there was dulness on percussion; this condition was not found to exist in any other part of the lungs. The breathing was insufficient and coarse, there was a peculiar resonance of the voice, an abnormal click-like sound when he spoke or coughed, a kind of sound more easily imagined than described. Broncophony and bronchial respiration could be heard, and differed from that of health by being distinctly audible near the humeral extremity of the clavicle. With such an array of symptoms there seemed but little grounds for doubting the existence of tubercular deposits. But still the evidence was not absolutely certain, yet the symptoms could not be distinguished from those which have been characterized incipient phthisis, which, in hundreds of cases, have not been interrupted, but passed on through all the different stages of the disease to a fatal termination.

Without making known to him or his family the character of the disease, I directed

Pil. Hyd. grs. VI.

Opii " I.

to be taken at bed time. In a few days I directed the following mixture:

Comp. Syrp. Sarsaparl. ℥ III.
Tct. Iodine, ʒ III.

A teaspoonful to be taken every six hours. Directed the use of the nitro-muriatic bath four times per week. He was also directed to use a nutritious diet, boiled pork and eggs in moderate quantities. He continued to use the Iodine without interruption for three months, during which time I used occasionally a Dover powder or dose of morphine to procure rest. Several weeks passed without any favorable change in the symptoms, but rather an aggravation. The expectoration became puriform, streaked occasionally with blood, his feet became edematous. Notwithstanding all this the remedy was continued, and part of the time given in larger doses. About the first of Feb., 1854, the unfavorable symptoms began gradually to disappear, and by the 15th of that month, which was about three months after he commenced the use of the Iodine, the expectoration and cough ceased, and he was so much improved in every respect that the remedy was discontinued, and he is now a hearty man for his age, without any perceivable symptoms of phthisis.

Case 2d.—On the first day of June, 1855, Mr. W. called at my office for medical advice; he was 34 years of age, and engineer on the Wabash Valley Railroad. He had nearly all the rational symptoms enumerated in the preceding case, but the physical signs did not clearly establish the truthfulness of those symptoms. He had cough, hectic fever, emaciation, pain in the left lung, but it was in the middle lobe, or near the nipple, cough troubled him very much while lying down and early in the morning. But still there was but little dullness on percussion, there was but little morbid sound when he spoke or coughed. With such symptoms, my diagnosis was incipient consumption, although the evidence was not conclusive. I ordered the same treatment as in the first case, but in a few weeks the cough left, night sweats subsided. He is a small man, but in three months under the above treatment he gained twenty-five pounds, and now enjoys good health.

I do not wish to be understood as pronouncing all cases of phthisis pulmonalis curable; facts will not justify such a conclusion. But that the disease has frequently been cured I do not entertain a doubt. The first and most important indication in the treatment of this disease is, to improve faulty nutrition. Codliver oil has gained much reputation in the cure of phthisis, but we incline to the belief that the virtue of this remedy may be attributed to the fact that it is easily assimilated, consequently its remedial effects are referable more to its nutritious than to its medicinal properties. Dr. Williams, in his Principles of Medicine, says he has "for years referred tubercle to a deranged condition of the nutritive material. In many cases they are found disseminated in so many textures, after few or no symptoms of inflammation, that it is imposible to regard them otherwise than as the result of modified textural nutrition. Dr. J. A. Bennett, of Edinburgh, in a work recently published, says—"We are warranted in drawing the conclusion, that if during the advance of phthisis pulmonalis, those means can be discovered which check further tubercular exudation and keep the strength and nutritive process of the economy such tubercular exudations as have occurred, will be rendered abortive, and that even large ulcerations will heal up and cicatrize. The important point, practically, is to ascertain what these means are, and how they may be put into operation." M. Piorry read a paper to the French Academy of Medicine on the 24th day of January, 1854, in which he recommended Iodine inhalations for the cure of phthisis. He refers to thirty-one cases treated by inhaling the tinct. and vapor of Iodine, twenty of whom were benefited, seven apparently cured, and four deaths without any amelioration. Dr. Wood, in his practice of Medicine, speaking in regard to the curability of consumption, says—"I am not one of those who believe that phthisis is in all cases necessarily fatal.—On the contrary, I believe that in one stage or another, it is occasionally cured, or at least ends in perfect recovery." With such testimony before us, we hope no physician will be so skeptical in regard to the cure of phthisis, as to reject all curative treatment. If the disease is curable, the profession owes it to humanity to seek out the remedy.

In the cure of *dyspepsia*, that congeries of disease, the prevailing malady of civilized life, I have found Iodine far superior to all other remedies. It is admissable in any form of the disease not attended with inflammation of the stomach. I have used it in a great variety of cases, with the most salutary results. It is best administered in the following formula:

| | |
|---|---|
| ℞ Sang. Canadensis, | ʒ I. |
| Podoph. Pil. Rad. | ʒ I. |
| Rad. Gentian, | ℥ I. |
| Hondoms Sarsaparilla, | ℥ I. |

Put in one pt. water, boil to ½ pt., strain and add,

| | |
|---|---|
| Tct. Iodine, | ℥ I. |
| Oil Sasafras, | gtt. XX. |
| Sach. Alba, | ℥ III. |
| Brandy, | ½ pt. |

Of this mixture I give from one to two drms. three or four times per day. Some may suppose that the benefit is derived from the articles combined with the Iodine in the above mixture, but this cannot be the case, from the fact that when the Iodine is left out, the mixture is comparatively worthless. I have used this remedy in scrofulous affections, amenorrhœa, dysmenorrhœa, and chronic diseases of the liver and spleen, with unequivocal benefit. In *nursing sore mouth* Iodide Potassium is far superior to all other remedies. I have used it with extraordinary benefit in many cases.

Time will not permit me to extend these remarks further, but it is hoped the hints herein presented, will induce others more competent than myself, to test more fully the curative properties of Iodine in the treatment of the diseases to which I have referred.

